# Similar graft rerupture rates after ACL reconstruction with or without lateral extra‐articular procedure in patients over 40 years

**DOI:** 10.1002/jeo2.70533

**Published:** 2025-11-05

**Authors:** Mohamad K. Moussa, Antoine Orso, Eugénie Valenti, Nicolas Lefèvre, Alain Meyer, Antoine Gerometta, Olivier Grimaud, Yoann Bohu, Alexandre Hardy

**Affiliations:** ^1^ Groupe Hospitalier Séléstat‐Obernai Séléstat France; ^2^ Clinique du sport, Ramsay Santé Paris France

**Keywords:** anterior cruciate ligament reconstruction (ACLR), lateral extra‐articular procedures (LEAP), graft failure, long‐term follow‐up

## Abstract

**Purpose:**

To compare the outcomes of isolated anterior cruciate ligament reconstruction (ACLR) versus ACLR+ lateral extra articular procedures (LEAP) at midterm follow up in patients older than 40 years old.

**Methods:**

This was a retrospective cohort study based on prospectively collected data of patients aged over 40 years who underwent ACLR, with or without LEAP, from 2012 to 2022. Patients were matched by age, sex, preinjury Tegner score, and follow‐up duration to minimize selection bias. LEAP was reserved for higher‐risk individuals—specifically those with high pivot shift, valgus alignment, recurvatum, chronic lesions, suturable meniscal tears, or high activity levels. The primary outcome was graft rerupture rate, defined as the recurrence of subjective instability associated with abnormal anterior laxity on clinical examination (Lachman grade ≥2 and/or pivot shift grade ≥2) and confirmed by magnetic resonance imaging or intraoperative findings. Secondary outcomes included return‐to‐sport (RTS) rates, time to RTS, and functional scores, including the International Knee Documentation Committee (IKDC) score, Knee injury and Osteoarthritis Outcome Score (KOOS), Anterior Cruciate Ligament–Return to Sport after Injury (ACL‐RSI) score, Lysholm score, and Tegner Activity Scale (TAS).

**Results:**

A total of 216 patients were analyzed (142 ACLR, 74 ACLR + LEAP) at a mean follow‐up of 59.6 versus 64.5 months respectively (*p* > 0.05). The mean age was 44.7 (SD = 3.4) versus 44.4 (SD = 3.5) years, respectively (*p* > 0.05). Graft rerupture rates were low in both groups, with no significant difference (2.8% in ACLR vs. 1.3% in ACLR + LEAP, *p* > 0.05). Functional outcomes and non‐graft‐rupture related reoperation rates were also comparable. Return to the same sport was reported in 54.9% of the ACLR group and 59.5% of the ACLR + LEAP group (*p* > 0.05), with similar time to RTS. A subgroup analysis of patients undergoing ACLR using hamstring grafts only showed no reruptures in the ACLR + LEAP group compared to a 2.8% rerupture rate in the ACLR alone group (*p* > 0.05).

**Conclusion:**

No significant reduction in overall graft rerupture risk was observed with the addition of LEAP to ACLR in patients over 40 years old. Functional outcomes and reoperation rates were comparable between groups.

**Study Design:**

Cohort study, level of evidence 3.

**Level of Evidence:**

Level III, cohort study.

## Graphical abstract

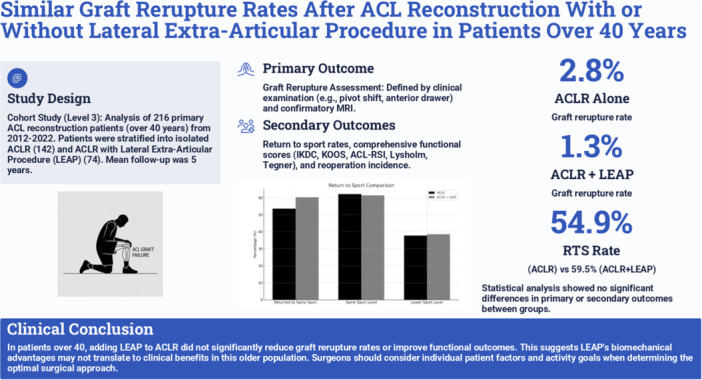

**Abbreviations**
ACLR: anterior cruciate ligament reconstruction
ACL‐RSI: ACL return to sport after injury
BMI: body mass index
BPTB: bone‐patellar tendon‐bone
IKDC: International Knee Documentation Committee
IQR: interquartile range
KOOS: Knee injury and Osteoarthritis Outcome Score
LEAP: lateral extra‐articular procedures
MRI: magnetic resonance imaging
RTS: return to sport
SD: standard deviation
TAS: Tegner Activity Scale

## INTRODUCTION

Anterior cruciate ligament reconstruction (ACLR) is a well‐established surgical procedure aimed at restoring stability and kinematics to the knee joint after an ACL injury [1, 8]. Traditionally, this procedure was primarily reserved for younger, more active populations [4, 20, 37]. However, with the growing participation of older individuals in sports and recreational activities, ACLR is increasingly being performed in patients over 40 years old, with the goal of improving knee function and stability in this age group [3, 29, 35].

With the growing number of ACLR procedures being performed annually, the incidence of rerupture is also on the rise [7, 10, 46]. Various graft options and surgical techniques have been explored to improve outcomes and reduce rerupture rates [6, 12, 24, 36, 41]. Among these, lateral extra‐articular procedures (LEAP) have gained attention, as an add‐on procedure, due to their ability to reduce rerupture rates, regardless of the graft type used—whether hamstring, bone‐patellar tendon‐bone (BPTB), or quadriceps tendon grafts [6, 10, 11, 12, 36, 41]. By restoring the mechanical functions of the anterolateral ligament, LEAP has been demonstrated to be as effective in paediatric populations, adults, athletes and even patients over 30 years of age [6, 12, 16, 24, 36, 41]. However, its relevance in the older age group and less active population is yet to be established. While many studies have demonstrated that less active patients over 40 years old may achieve satisfactory outcomes without ACLR, this does not apply universally [16]. A subset of patients—those with persistent instability despite conservative management—continue to experience functional limitations that warrant surgical intervention. In these cases, especially when risk factors for graft rerupture are present (e.g., high pivot shift, valgus alignment, recurvatum, meniscal lesions), the addition of a LEAP may be considered [10, 11, 24, 27]. Pettinari et al. demonstrated a reduced failure rate with LEAP even in patients over 30 years of age, suggesting that age alone should not preclude consideration of lateral augmentation when indicated by individual risk factors [27]. On the other hand, a recent systematic review found that patients in this age group generally have low rates of rerupture following ACLR, with a rerupture rate of approximately 2.7% at 2 years postsurgery [29, 31]. This lower rate questions the benefits of LEAP in this age group (over 40 years old).

The aim of this study was to compare the rerupture rate in patients aged >40 years when treated by ACLR with or without LEAP. The secondary aim was to compare the functional outcomes of these 2 populations.

The study hypothesized that the addition of LEAP to ACLR would reduce the graft rerupture rate in patients older than 40 years. Additionally, it hypothesized that functional outcomes would be superior in patients undergoing ACLR combined with LEAP compared to ACLR alone.

## MATERIALS AND METHODS

### Study design and inclusions

This was a retrospective cohort study targeting patients who underwent ACLR between 2012 and 2022. The study received approval from the institutional review board, and all patients provided written informed consent at the time of initial inclusion in the cohort. The data belong to the French Prospective Anterior Cruciate Ligament Reconstruction Cohort Study (FAST); Registration Number: NCT02511158.

Inclusion criteria were all consecutive patients aged over 40 years who underwent ACLR with or without LEAP, regardless of the technique used. Patients were required to have complete preoperative and postoperative functional outcome data (IKDC, KOOS, ACL‐RSI, Lysholm, Tegner), a minimum follow‐up of 2 years, and clinical assessment of graft integrity.

Exclusion criteria included multi‐ligamentous injury, revision surgery, age <40 years, knee osteoarthritis classified as grade 2 or higher according to the Ahlbäck radiographic classification, refusal to participate.

### Surgical technique and postoperative Rehabilitation Protocol

Seven surgeons, specialized in sports surgery, operated on patients included in the study. Several graft types were involved including hamstring grafts [19, 28], bone‐patellar‐tendon‐bone grafts, and modified Macintosh grafts [9]. All reconstructions were performed using autografts; no allografts were used in this study.

The choice of LEAP type was based on surgeon preference with options including anterolateral ligament (ALL) reconstruction [33, 34], lateral extraarticular tenodesis [47], and as part of the Modified Macintosh technique.

The choice of employing the LEAP procedure was based on several criteria related to high pivot shift instability, valgus knee alignment, patient demand, recurvatum, chronicity of the lesion, presence of suturable meniscal lesion and type and level of the patient's sports activity.

### Outcome measures

The primary outcome measure was the rate of rerupture. This diagnosis required recurrent subjective instability reported by the patient, combined with positive objective findings on physical examination, defined as a Lachman test graded 2 or 3 (increased anterior tibial translation with a soft endpoint) and/or a pivot‐shift test graded 2 or higher (glide or clunk). All clinical assessments were performed by surgeons themselves. Graft rupture was systematically confirmed by MRI, demonstrating graft discontinuity or non‐visualization of graft fibres. Cases with an elongated but continuous graft without clinical instability were not classified as rerupture. Only patients with both clinical instability and MRI‐confirmed graft rupture were considered as rerupture cases.

Secondary outcome measures included various metrics related to return‐to‐sports (RTS), such as the rate of RTS, the time taken to return to sport, and the quality of the RTS. The quality of RTS was assessed based on the patient's perception of their performance relative to their preoperative status, categorized as either the same or lower than before surgery.

Additional outcome measures encompassed functional scoring systems. These included the International Knee Documentation Committee (IKDC) score [13], the Knee injury and Osteoarthritis Outcome Score (KOOS) [25, 30] with subcategories for Pain, Symptoms, Activities of Daily Living, Sport and Recreation, and Quality of Life. Other tools used for evaluation were the Tegner Activity Scale (TAS) [44], the ACL Return to Sport after Injury (ACL‐RSI) [2, 45] scale, and the Lysholm score [21].

A subgroup analysis was conducted specifically in patients undergoing ACLR with hamstring grafts, comparing outcomes between those with and without LEAP.

### Data collection

Data were collected prospectively by the surgeons and fellows using the online software Websurvey (Paris, France). Patients were invited to complete standardized questionnaires at 1, 2, 4 and 6 years postoperatively. Patients accessed the software to input demographic information and patient‐reported outcomes, while surgeons and fellows used it to record physical examination findings, technical details, objective outcome measures, and complications. In cases of missing data, patients were contacted and reviewed to ensure the information was complete. For graft rerupture assessment, all patients were seen in physical consultation, where detailed clinical examination was performed. In cases of suspected rerupture, MRI confirmation and surgical findings were used to validate the diagnosis.

### Statistical analysis

The study population was matched using a propensity score to minimize selection bias between the two reconstruction technique groups. Participants were matched at a 1:2 ratio, using a logit scale with a caliper width of 0.2. The matched variables included age, sex, preinjury TEGNER score, presence of meniscal lesions, and follow‐up duration.

The required sample size was estimated based on the assumption that a 10% difference in graft rupture rates would be observed between groups based on the results of Moussa et al. [24], assuming that ACLR + LEAP group in this study is a high‐risk group based on the indication criteria for this cohort. With a Type I error risk of 5.0%, a Type II error risk of 20.0%, and a one‐sided test, it was calculated that 72 patients would be needed in each group, for a total of 144 patients.

Qualitative variables, described by their frequency and percentage, were compared using Pearson's Chi‐squared test or Fisher's exact test, depending on group sizes. Quantitative data were described by means and standard deviations, and comparisons were made using appropriate statistical tests, either Student's *t*‐test or the Mann–Whitney test.

A *p* < 0.05 was considered statistically significant. All statistical analyses were performed using R software (version 4.2).

## RESULTS

### Participants and sample size (Figure 1)

**Figure 1:**
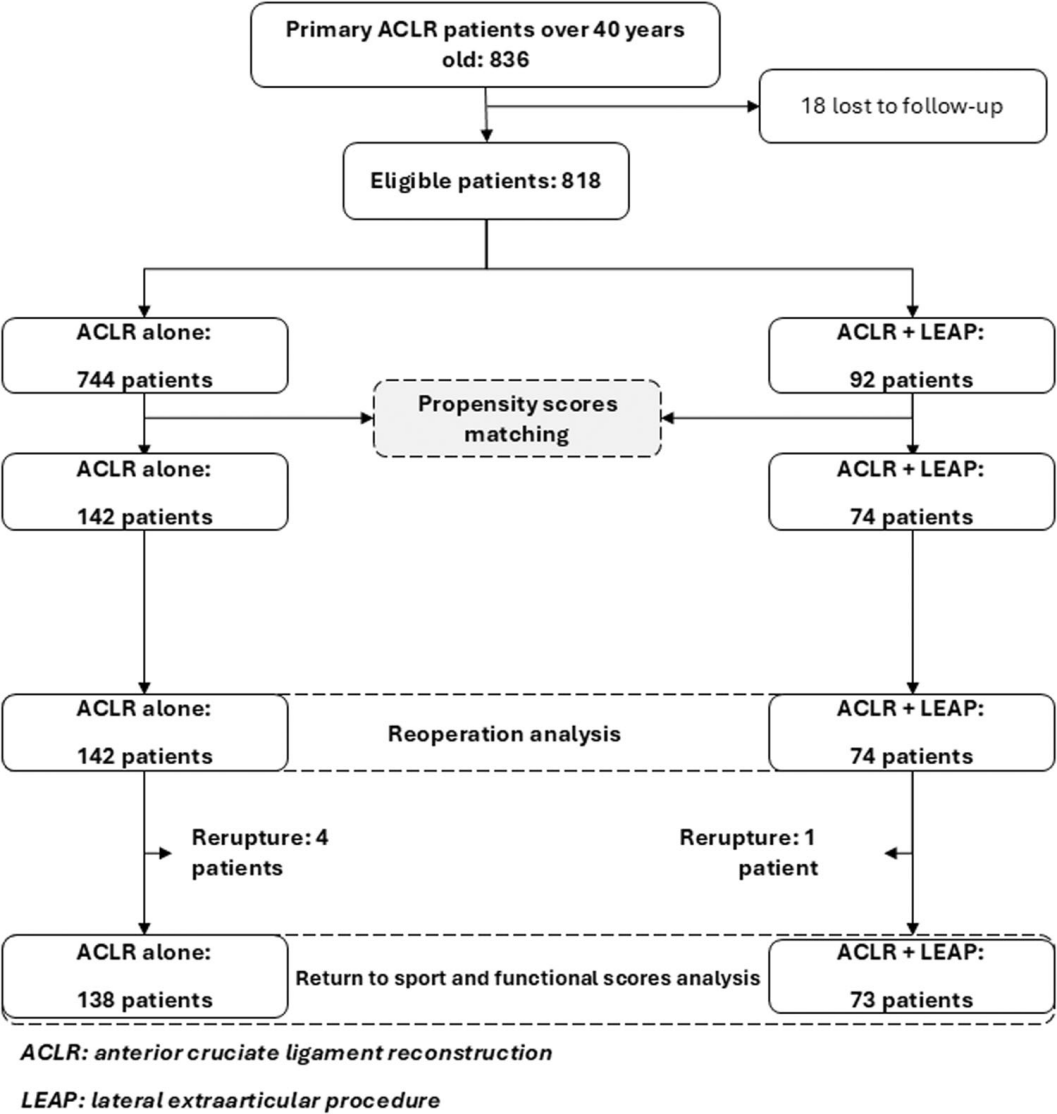
Flow chart.

During the study period, 836 patients ≥40 years underwent primary ACLR. After excluding 18 lost to follow‐up, 92 underwent ACLR + LEAP and 744 underwent ACLR alone. The application of matching at a ratio of 2:1 generated two groups: the ACLR group with 142 patients and the ACLR + LEAP group with 74 patients (6 patients had only 1 match). The groups had similar characteristics: age (44.7 years, SD = 3.4 vs. 44.4 years, SD = 3.5 years), and BMI (*p* > 0.05). Time from accident to surgery was significantly longer in the ACLR + LEAP group (10.1 vs. 6.3 months, *p* = 0.02).

Preoperative functional scores, including IKDC, KOOS sub‐scores, and Lysholm, were similar between the groups (all *p* > 0.05). (Table 1).

**Table 1:** Patient's characteristics.

	ACLR alone	ACLR + LEAP	*p*‐Value
Number of participants (*N*)	142	74	
Age (years) on the day of operation, mean (SD) [min‐max]	44.7 (3.4) [40.2;57.6]	44.4 (3.5) [40.0;54.7]	>0.05
BMI (kg/m^2^), mean (SD)	25.4 (3.2)	25.7 (2.9)	>0.05
Sex, *N* (%)			>0.05
Women	25 (17.6%)	13 (17.6%)	
Men	117 (82.4%)	61 (82.4%)	
Knee, *N* (%)			>0.05
Right	77 (54.2%)	37 (50.0%)	
Left	65 (45.8%)	37 (50.0%)	
Circumstance of accident, *N* (%)			>0.05
Public road accident	4 (2.8%)	1 (1.4%)	
Work accident	4 (2.8%)	2 (2.7%)	
Domestic accident	65 (92.9%)	66 (94.3%)	
Sports accident	127 (89.4%)	70 (94.6%)	
Level of sports practice, *N* (%)			>0.05
Professional	0 (0.0%)	2 (2.7%)	
Competition	30 (21.1%)	19 (25.7%)	
Regular leisure	93 (65.5%)	42 (56.8%)	
Occasional leisure (active)	19 (13.4%)	11 (14.9%)	
Type of sport, *N* (%)			**0.0419**
Pivot contact (Football, handball, rugby, basketball, judo)	64 (45.1%)	36 (48.6%)	
Pivot without contact (tennis, skiing, badminton, volleyball)	46 (32.4%)	31 (41.9%)	
Without pivot (jogging, biking, swimming)	32 (22.5%)	7 (9.5%)	
Chondral lesion, *N* (%)			>0.05
No	92 (64.8%)	44 (59.5%)	
Yes	50 (35.2%)	30 (40.5%)	
Meniscus injury, *N* (%)			
Any type	88 (62.0%)	46 (62.2%)	>0.05
Medial meniscus injury	64 (45.1%)	39 (52.7%)	>0.05
Lateral meniscus injury	40 (28.2%)	19 (25.7%)	>0.05
Type of ACL reconstruction			<0.0001
Hamstring‐based graft	139 (97.9%)	42 (56.8%)	
Bone‐patellar‐tendon Bone graft	3 (2.1%)	8 (8.1%)	
Modified Macintosh graft	0 (0.0%)	26 (35.1%)	
Delay accident‐surgery in months, median (IQR)	6.3 (3.4;20.9)	10.1 (4.3;66.4)	**0.0295**
Follow‐up in months, mean (SD)	59.6 (26.9)	64.5 (25.5)	>0.05
	Min–Max = 24–116.3	Min‐Max = 24 ‐117.6	
Preoperative functional scores			
IKDC score, mean (SD)	58.2 (18.3)	60.7 (14.4)	>0.05
KOOS			
Symptom and stiffness score, mean (SD)	72.0 (19.0)	76.2 (17.4)	>0.05
Pain score, mean (SD)	73.1 (17.6)	77.2 (14.6)	>0.05
Daily life score, mean (SD)	80.7 (18.2)	84.2 (14.3)	>0.05
Sport score, mean (SD)	42.5 (27.7)	46.4 (23.3)	>0.05
Quality of life score, mean (SD)	26.6 (19.4)	25.7 (18.2)	>0.05
Lysholm, mean (SD)	69.0 (17.4)	72.8 (13.4)	>0.05
Tegner activity scale (out of 10), mean (SD)	6.8 (1.5)	6.9 (1.6)	>0.05
ACL‐RSI, mean (SD)	33.5 (29.7)	38.4 (30.0)	>0.05

Abbreviations: ACLR, anterior cruciate ligament reconstruction; BMI, body mass index; IQR, interquartile range; LEAP, lateral extra‐articular procedure; SD, standard deviation.

### Rate of rerupture and other complications (Table 2 and Figure 2)

**Table 2:** Rate of rerupture and other complications in both group.

Complications	ACLR alone (*n* = 142)	ACLR + LEAP (*n* = 74)	*p* Value
Graft rupture	4 (2.8%)	1 (1.3%)	>0.05
Time to rerupture (median)	18 (10–768)	6	N/A
Nongraft rupture related reoperation^a^	7 (4.9%)	3 (4.1%)	>0.05
Arthrolysis or cyclops	3 (42.9%)	0 (0%)	>0.05
Meniscectomy	1 (14.3%)	3 (100%)	>0.05
Chondroplasty	1 (14.3%)	0 (0%)	>0.05
Haematoma evacuation	1 (14.3%)	0 (0%)	>0.05
Mobilization under general anaesthesia	1 (14.3%)	0 (0%)	>0.05
Mean time to revision in months (SD)	16.0 (22.0)	26.4 (28.9)	>0.05

Abbreviations: ACLR, anterior cruciate ligament reconstruction; LEAP, lateral extra‐articular procedure; NA, not applicable; SD, standard deviation.

a There may be more than one cause per revision, the percentage corresponds to the number of events over the total number of revisions in each group.

**Figure 2:**
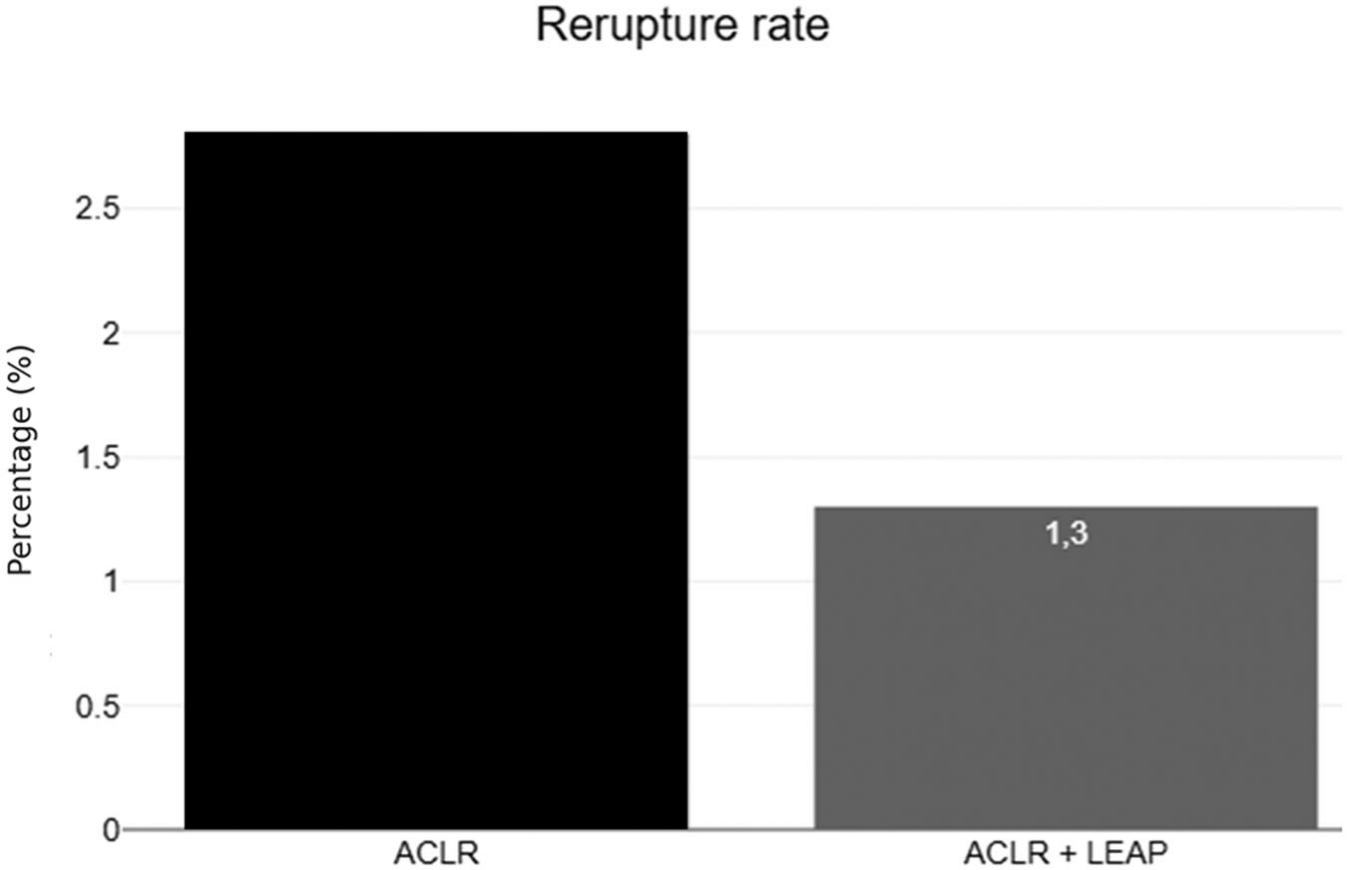
Graft rerupture rate as compared between isolated ACL reconstruction (ACLR) and ACLR combined with a lateral extra‐articular procedure (LEAP). Both groups demonstrated low rerupture rates.

Graft rupture rates were not statistically different between groups, with 2.8% (4/142) in the ACLR alone group and 1.3% (1/74) in the ACLR + LEAP group (*p* > 0.05).

Nongraft rupture related reoperation rates were also comparable, occurring in 4.9% of the ACLR group and 4.1% of the ACLR + LEAP group (*p* > 0.05). The mean time to revision surgery was longer for ACLR + LEAP (26.4 ± 28.9 months) than for ACLR alone (16.0 ± 22.0 months), though not significantly different (*p* > 0.05, Table 2, Figure 2).

### Functional outcomes (Table 3 and Figure 3)

**Table 3:** Functional outcomes.

	ACLR alone (*n* = 138)	ACLR + LEAP (*n* = 73)	*p*‐Value
IKDC Score: Mean (SD)	81.4 (13.9)	81.6 (14.3)	>0.05
KOOS: Mean (SD)			
KOOS Pain Score	90.7 (12.0)	90.6 (12.3)	>0.05
KOOS Symptoms Score	86.4 (13.5)	86.6 (15.6)	>0.05
KOOS Daily Life Score	94.9 (9.5)	94.6 (10.0)	>0.05
KOOS Sport Score	79.7 (21.9)	79.5 (23.1)	>0.05
KOOS Quality of Life Score	76.0 (25.0)	73.7 (27.9)	>0.05
Tegner Activity Scale (out of 10): Mean (SD)	5.2 (1.7)	5.0 (1.9)	>0.05
ACL‐RSI Score: Mean (SD)	66.2 (26.2)	66.2 (31.4)	>0.05
Lysholm Score: Mean (SD)	86.0 (15.9)	89.5 (12.1)	>0.05
Return to the same sport, *N* (%)	74 (53.6%)	44 (60.3%)	>0.05
If yes, time to return (months)	6.3 (6.1)	6.8 (6.2)	>0.05
Sport level compared to preinjury			>0.05
* Lower*	28 (37.8%)	17 (38.6%)	>0.05
* Same*	46 (62.2%)	27 (61.4%)	

Abbreviations: ACLR, anterior cruciate ligament reconstruction; ACL‐RSI, Anterior Cruciate Ligament–Return to Sport after Injury; IKDC, International Knee Documentation Committee; KOOS, Knee Injury and Osteoarthritis Outcome Score; LEAP, lateral extra‐articular procedure; SD, standard deviation.

**Figure 3:**
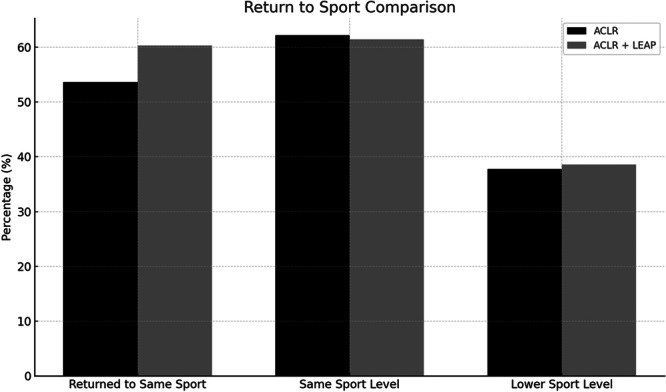
Return‐to‐sport outcomes as compared between ACLR and ACLR + LEAP. Metrics include return to the same sport, return at the same sport level, and return at a lower sport level. ACLR, anterior cruciate ligament reconstruction; LEAP, lateral extra‐articular procedure.

All functional scores were similar between groups (*p* > 0.05). Return to the same sport was reported by 74 (53.6%) of the ACLR group and 44 (60.3%) of the ACLR + LEAP group (*p* > 0.05), with no significant difference in the time to return (6.3 vs. 6.8 months, *p* > 0.05, Table 3, Figure 3).

### Subgroup analysis of patients with ACLR using hamstring graft only with or without LEAP (Tables 4, 5, 6)

**Table 4:** Demographic characteristics of patients with ACLR using hamstring graft only with or without LEAP.

	ACLR alone	ACLR + LEAP	*p*‐Value
Number of participants (*N*)	139	42	
Age on the day of operation, mean (SD) [min‐max]	44.7 (3.4) [40.2; 57.6]	44.7 (3.7) [40.1; 54.7]	>0.05
BMI, mean (SD)	25.4 (3.2)	25.7 (3.1)	>0.05
Sex, *N* (%)			>0.05
Women	25 (18.0%)	12 (28.6%)	
Men	114 (82.0%)	30 (71.4%)	
Knee, *N* (%)			>0.05
Right	75 (54.0%)	21 (50.0%)	
Left	64 (46.0%)	21 (50.0%)	
Circumstance of accident, *N* (%)			>0.05
Public road accident	4 (2.9%)	1 (2.4%)	
Work accident	7 (5.0%)	1 (2.4%)	
Domestic accident	4 (2.9%)	1 (2.4%)	
Sports accident	124 (89.2%)	39 (92.9%)	
Level of sports practice, *N* (%)			>0.05
Professional	0 (0.0%)	1 (2.4%)	
Competition	29 (20.9%)	8 (19.0%)	
Regular leisure	91 (65.5%)	26 (61.9%)	
Occasional leisure (active)	19 (13.6%)	7 (16.7%)	
Type of sport, *N* (%)			>0.05
Pivot contact (Football, handball, rugby, basketball, judo)	63 (45.3%)	19 (45.2%)	
Pivot without contact (tennis, skiing, badminton, volleyball)	45 (32.4%)	19 (45.2%)	
Without pivot (jogging, biking, swimming)	31 (22.3%)	4 (9.5%)	
Chondral lesion, *N* (%)			>0.05
No	91 (65.5%)	23 (54.8%)	
Yes	48 (34.5%)	19 (45.2%)	
Meniscus injury, *N* (%)			
Any type	85 (61.2%)	30 (71.4%)	>0.05
Internal meniscus injury	63 (45.3%)	27 (64.3%)	0.0355
External meniscus injury	38 (27.3%)	12 (28.6%)	>0.05
Delay accident‐surgery in months, median (IQR)	6.3 (3.4;21.1)	23.7 (6.6;104.1)	0.0003
Follow‐up in years, mean (SD)	59.3 (26.7)	66.3 (28.7)	>0.05
Preoperative functional scores			
IKDC score, mean (SD)	57.9 (18.3)	62.2 (14.1)	>0.05
KOOS			
Symptom and stiffness score, mean (SD)	71.7 (19.0)	80.0 (14.4)	0.01
Pain score, mean (SD)	73.0 (17.6)	80.2 (13.6)	0.02
Daily life score, mean (SD)	80.5 (18.4)	85.9 (12.8)	>0.05
Sport score, mean (SD)	42.1 (27.5)	45.8 (23.5)	>0.05
Quality of life score, mean (SD)	26.3 (19.5)	23.5 (17.4)	>0.05
Lysholm, mean (SD)	68.4 (17.3)	73.4 (12.5)	>0.05
Tegner activity scale (out of 10), mean (SD)	6.8 (1.5)	6.7 (1.7)	>0.05
ACL‐RSI, mean (SD)	33.7 (29.5)	31.3 (27.8)	>0.05

Abbreviations: ACLR, anterior cruciate ligament reconstruction; BMI, body mass index; IQR, interquartile range; LEAP, lateral extra‐articular procedure; SD, standard deviation.

**Table 5:** Complication in the subgroup analysis of patients with ACLR using hamstring graft only with or without LEAP.

Complications	ACLR alone (*n* = 139)	ACLR + LEAP (*n* = 42)	*p* Value
Graft rupture	4 (2.8%)	0 (0%)	>0.05
Time to rerupture (Median)	18 (10‐768)	‐	N/A
Nongraft rupture‐related reoperation^a^	7 (5.0%)	1 (2.4%)	>0.05
Arthrolysis or cyclops	3 (42.9%)	0 (0%)	>0.05
Meniscectomy	1 (14.3%)	1 (14.3%)	>0.05
Chondroplasty	1 (14.3%)	0 (0%)	>0.05
Haematoma evacuation	1 (14.3%)	0 (0%)	>0.05
Mobilization under general anaesthesia	1 (14.3%)	0 (0%)	>0.05
Mean time to revision in months (SD)	16.0 (22.0)	12.4 (17.9)	>0.05

Abbreviations: ACLR, anterior cruciate ligament reconstruction; LEAP, lateral extra‐articular procedure; N/A, not applicable; SD, standard deviation (SD).

a There may be more than one cause per revision, the percentage corresponds to the number of events over the total number of revisions in each group.

**Table 6:** Functional outcomes in the subgroup analysis of patients with ACLR using hamstring graft only with or without LEAP.

	ACLR alone (*n* = 135)	ACLR + LEAP (*n* = 42)	*p*‐Value
IKDC Score: Mean (SD)	81.4 (13.8)	81.1 (14.0)	>0.05
KOOS: Mean (SD)			
KOOS Pain Score	90.7 (12.0)	89.8 (12.5)	>0.05
KOOS Symptoms and Stiffness Score	86.2 (13.6)	85.6 (15.9)	>0.05
KOOS Daily Life Score	94.8 (9.6)	94.6 (10.3)	>0.05
KOOS Sport Score	79.6 (22.1)	78.8 (21.4)	>0.05
KOOS Quality of Life Score	75.9 (25.2)	72.0 (27.7)	>0.05
Tegner Activity Scale (out of 10): Mean (SD)	5.2 (1.7)	4.8 (1.8)	>0.05
ACL‐RSI Score: Mean (SD)	66.0 (26.3)	63.8 (29.2)	>0.05
Lysholm Score: Mean (SD)	85.9 (16.0)	89.2 (12.7)	>0.05
Return to the same sport, *N* (%)	72 (53.3%)	23 (54.8%)	>0.05
If yes, time to return (months)	6.3 (6.1)	6.0 (6.6)	>0.05
Sport level compared to preinjury			>0.05
Lower	27 (37.5%)	11 (47.8%)	>0.05
Same	45 (62.5%)	12 (52.2%)	

Abbreviations: ACLR, anterior cruciate ligament reconstruction; ACL‐RSI, anterior cruciate ligament–return to sport after injury; IKDC, International Knee Documentation Committee; LEAP, lateral extra‐articular procedure; KOOS, Knee injury and Osteoarthritis Outcome Score; SD, standard deviation; TAS, Tegner Activity Scale.

Demographic characteristics in the subgroup analysis were comparable between groups except for a significantly longer delay between injury and surgery in the ACLR + LEAP group (median delay: 23.7 months [IQR: 6.6–104.1] vs. 6.3 months [IQR: 3.4–21.1], *p* = 0.0003), and higher preoperative KOOS scores in the ACLR + LEAP group regarding symptoms and stiffness (80.0 ± 14.4 vs. 71.7 ± 19.0, *p* = 0.0182) and pain (80.2 ± 13.6 vs. 73.0 ± 17.6, *p* = 0.0270, Table 4). Functional outcomes were similar between groups, with comparable return‐to‐sport outcomes (53.3% in ACLR alone vs. 54.8% in ACLR + LEAP; *p* > 0.05, Table 5). However, there were no reruptures observed compared to a 2.8% rerupture rate in the ACLR alone group (*p* > 0.05, Table 6).

## DISCUSSION

The main finding of this article was that the addition of LEAP is not associated with statistically significant decreased risk of graft rerupture in patients over 40‐years‐old undergoing ACLR.

Although not statistically significant, this finding is particularly relevant because LEAP was specifically indicated for patients considered at higher baseline risk of rerupture. These included individuals with high pivot shift instability, valgus knee alignment, patient functional demand, recurvatum, chronicity of the lesion, presence of suturable meniscal lesion and type and level of the patient's sports activity. In this context, the absence of rerupture in the higher‐risk LEAP group, despite the lack of statistical difference, may be viewed as a signal of potential protective benefit. However, study was not powered to detect differences in this subgroup; therefore, this observation should be interpreted cautiously and regarded as hypothesis‐generating. This aligns with previous literature that highlights the benefits of LEAP in reducing rerupture risk across most age groups [6, 11, 12, 14, 22, 24, 27, 32, 36, 39, 40, 41]. For example, Pettinari et al. reported a significant reduction in graft rerupture with LEAP in patients over 30 years, demonstrating a 3‐fold lower risk of rerupture compared to isolated ACLR (*p* = 0.0116) [27]. However, most of these studies have focused on younger and more active populations, where the baseline risk of rerupture is generally higher [6, 11, 12, 24, 27, 36, 41]. A recent systematic review and meta‐analysis of randomized controlled trials by Kan et al., which included 10 high‐quality RCTs comparing ACLR with and without LEAP [14]. This analysis demonstrated that the addition of LEAP significantly reduced graft rupture rates (risk ratio, 0.21 [95% CI, 0.08–0.55]; *p* < 0.001) and rotatory instability, without sustained adverse effects beyond 6 months [14].

A key explanation for the lack of significant differences in our overall cohort could be the inherently lower baseline risk of graft rerupture in older populations. Systematic reviews, such as those by Tan et al. and Robert et al., have shown that patients over 40 or even 50 years old experience low rates of graft rerupture, around 2.7% or lower [29, 43]. For instance, Tan et al. found only 1 rerupture out of 287 patients over 50 years old across four studies [43]. This lower baseline risk may contribute to the lack of significant differences in outcomes between ACLR and ACLR + LEAP, as the potential for further risk reduction is limited. In contrast, studies involving younger populations show a significant benefit of LEAP [11, 18, 24]. Moussa et al. demonstrated up to 6‐fold increase in rerupture risk for patients under 18 who did not receive LEAP, indicating that in higher‐risk groups, the benefit of LEAP is more pronounced [24]. Similarly, Pettinari et al. also found a higher risk of rerupture in the younger age bracket (30–35 years), further supporting the age‐related variability in outcomes [24].

Another consideration is that, for older patients with low activity, conservative management has been shown to be effective, and is sometimes considered the first‐line approach in this population. For example, Hellberg et al. demonstrated favourable outcomes with conservative treatment in patients over 30 years old with low activity levels [16]. However, it is important to note that in our study, the patients who underwent surgery were operated on late after their rupture, following the failure of conservative treatment to provide adequate stability to the knee.

The indication for LEAP should primarily be driven by the patient's phenotype and their associated risk of graft rerupture. Patients identified as high risk are likely to benefit most from the addition of LEAP. Recent reviews have identified key risk factors for graft rerupture, notably younger age (especially under 20), smaller graft diameter, lack of LEAP in adults, greater posterior tibial slope, use of allografts over autografts, and early return to high‐level sports [7, 10, 46]. When using allograft, the rate of rerupture in patients aged >40 years old is 8% as shown by Sylvia et al. [42].

Concerning functional outcomes, our data showed no significant differences between groups, aligning with findings from other studies in literature [5, 15, 18, 23, 26, 38].

The rate of nongraft‐rupture related reoperations in our study was 5.6%, similar to that reported in the literature [17, 27]. This rate is lower than that observed in paediatric populations, where Moussa et al. reported 10.6%, and Monaco et al. found a similar rate of 10.8% [23, 24].

### Limitation

The study's limitations include its retrospective design and lack of randomization. Although the study spanned a long period and was conducted in a specialized sports surgery centre, the sample size remained limited. This was largely due to the relatively rare use of LEAP in this age group. As a result, the study is underpowered to detect small differences in graft rerupture rates, particularly given the low baseline rerupture rate observed in both groups. Additionally, although the matching process was implemented to minimize selection bias, it cannot fully eliminate the influence of unmeasured confounding variables. A key limitation of our study is indication bias, as LEAP was selectively performed in patients with higher baseline risk factors (e.g., high pivot shift, valgus, recurvatum, meniscal lesions). This non‐randomized selection may have confounded comparisons, potentially underestimating the protective effect of LEAP.

## CONCLUSION

Our study found no significant reduction in the overall graft rerupture risk with the addition of LEAP to ACLR in patients over 40 years old. Functional outcomes and reoperation rates remained similar between groups.

## AUTHOR CONTRIBUTIONS

*Study design and conceptualization*: Mohamad K. Moussa, Antoine Orso, Eugénie Valenti, Nicolas Lefèvre, Alain Meyer, Antoine Gerometta, Yoann Bohu, Olivier Grimaud, Alexandre Hardy. o *Data acquisition*: Mohamad K. Moussa, Antoine Orso, Yoann Bohu, Antoine Gerometta, Alexandre Hardy. o *Data review and analysis*: Mohamad K. Moussa, Antoine Orso, Antoine Gerometta, Alexandre Hardy. o *Manuscript review*: Mohamad K. Moussa, Antoine Orso, Yoann Bohu, Antoine Gerometta, Olivier Grimaud, Alexandre Hardy. o *Statistical analysis and table preparation*: Eugénie Valenti. o *Database creation and implementation*: Mohamad K. Moussa, Yoann Bohu, Alexandre Hardy, Nicolas Lefèvre. o *Manuscript draughting and guarantor role*: Mohamad K. Moussa.

## CONFLICT OF INTEREST STATEMENT

Nicolas Lefèvre: Consultant for Websurvey Society, Paris France/Alexandre Hardy: Consultant for Arthrex and Depuy.

## ETHICS STATEMENT

The study received approval from the institutional review board, and all patients provided written informed consent at the time of initial inclusion in the cohort. The data belong to the French Prospective Anterior Cruciate Ligament Reconstruction Cohort Study (FAST); Registration Number: NCT02511158.

## Data Availability

Data are available upon reasonable request.
